# Remarkable Response to Odevixibat in an Adult With Progressive Familial Intrahepatic Cholestasis Type 1 and Intractable Pruritus

**DOI:** 10.14309/crj.0000000000002033

**Published:** 2026-03-02

**Authors:** Fernando Gil-Lopez, Lydia A. Mercado, Nicole M. Loo

**Affiliations:** 1 Department of Internal Medicine, Mayo Clinic, Jacksonville, FL; 2 Clinical Trials Beyond Walls, Department of Research, Mayo Clinic, Jacksonville, FL; 3 Division of Hepatology and Liver Transplant, Department of Transplant, Mayo Clinic, Jacksonville, FL

**Keywords:** cholestasis, pruritus, bile acids

## Abstract

We report a case of significant clinical improvement after starting odevixibat in a 27-year-old patient with refractory pruritus due to progressive familial intrahepatic cholestasis type 1. He had poor response to multiple therapies and experienced profound decline in quality of life, describing his symptoms as emotionally exhausting and difficult to endure. He was evaluated for liver transplantation in March 2020 but was denied due to preserved liver function. Odevixibat was started in November 2021, resulting in biochemical improvement and dramatic sustained resolution of pruritus. He resumed normal activities, achieving excellent quality of life.

## INTRODUCTION

Progressive familial intrahepatic cholestasis (PFIC) refers to a group of rare autosomal recessive disorders characterized by impaired bile secretion, typically presenting in infancy or childhood with intrahepatic cholestasis.^1,2^ Clinical manifestations vary from early-onset liver disease to intermittent cholestasis triggered by external factors.^3^ PFIC type 1 is caused by mutations in the *ATP8B1* gene, which encodes FIC1, an ATPase involved in aminophospholipid translocation.^4^ Despite its role in bile formation, patients often have low or normal gamma-glutamyl transferase levels.^5^ We report the case of a male patient diagnosed with PFIC type 1 during adolescence, who suffered from severe pruritus and cholestasis refractory to conventional therapies. His case highlights the potential role of odevixibat, an ileal bile acid transporter inhibitor, in adult PFIC management.

## CASE REPORT

A 23-year-old man with genetically confirmed PFIC type 1 (*ATP8B1* homozygous mutation c.1982T>C) presented to our emergency department in May 2019 with jaundice, severe itching, vomiting, and abdominal pain. He had been diagnosed at age 17 following a liver biopsy and genetic testing. The biopsy showed mild intrahepatic and intracanalicular cholestasis, minimal iron deposition, and no fibrosis. Hepatic venous pressure gradient was measured at 14 mm Hg, consistent with clinically significant portal hypertension.

He also had bilateral tinnitus and severe sensorineural hearing loss, but no pancreatitis or chronic diarrhea. He reported fatigue, anorexia, and a 30-pound weight loss over 3 months. At presentation, he was taking cholestyramine, ursodiol, hydroxyzine, and occasional rifampin. Physical examination was notable for jaundice and excoriations. No fever, asterixis, or portal hypertension stigmata were documented.

Laboratory test results revealed total bilirubin (TB) 21.0 mg/dL, direct bilirubin 15.4 mg/dL, aspartate aminotransferase (AST) 37 U/L, alanine aminotransferase (ALT) 29 U/L, alkaline phosphatase (AP) 225 U/L, albumin 4.0 g/dL, sodium 136 mmol/L, international normalized ratio (INR) 1.0, leukocytes 5.3 × 10^9^/L, hemoglobin 14.5 g/dL, platelets 416 × 10^9^/L. Model for end‑stage liver disease (MELD)-Na was 18.

Abdominal ultrasound and transient elastography were normal. Abdominal MRI with contrast demonstrated no biliary obstruction and normal liver morphology. Hepatology was consulted, and Rifampin 150 mg twice daily was added, with partial, transitory improvement of his symptoms.

In March 2020, he was referred for liver transplant evaluation due to persistent pruritus and previous portal hypertension. His liver tests returned to normal at that time. Hepatitis A, B, and C, anti-mitochondrial antibodies, and anti-smooth muscle antibodies were negative. Serum ferritin 68 mcg/L, gamma-glutamyl transferase 7 U/L, ceruloplasmin 23 mcg/dL, iron 73 mcg/dL, alpha 1 antitrypsin (A1AT) 145 mg/dL, A1AT phenotype MM, and antinuclear antibodies 2.9 U (weak positive) were also tested.

He was denied liver transplant by the Transplant Committee on December 2020 due to preserved liver function (TB 0.4, direct bilirubin 0.1, AST 17, ALT 14, AP 70, albumin 4.6, INR 1.1, platelet count 294) and low MELD score (MELD-Na 6). Screening endoscopy in December 2020 showed no varices. Serum bile acids determination (October 2021) revealed total cholic acid >269.33 nmol/mL (≤5.0), total chenodeoxycholic acid 69.37 nmol/mL (≤6.0), total deoxycholic acid 0.78 nmol/mL (≤6.0), total ursodeoxycholic acid >236.76 nmol/mL (≤2.0), total bile acids >576.24 nmol/mL (≤19.0).

In November 2021, odevixibat was started at 2,800 mcg daily. Baseline laboratory test results showed TB 15.9 mg/dL, AP 163 U/L, AST 36 U/L, ALT 33 U/L. Within 2 weeks, he reported significant improvement in pruritus and bilirubin, with only transient diarrhea as a side effect. He continued ursodiol, hydroxyzine, sertraline, cholestyramine, and rifampin.

At four-week follow-up, TB was 1.6 mg/dL, AP 69 U/L, and transaminases normalized (Table 1). He transitioned to local gastroenterology care, with periodic laboratory test results reviewed by our hepatology team.

**Table 1.** Liver tests before and after initiation of odevixibat

	November 3, 2021	December 8, 2021	January 7, 2022	July 22, 2022	August 25, 2023	August 27, 2024	July 29, 2025
Total bilirubin (mg/dL)	15.9	2.8	1.0	0.3	0.3	0.5	0.5
Alkaline phosphatase (U/L)	163	64	64	65	82	69	58
Aspartate aminotransferase (U/L)	36	31	13	14	21	18	20
ALT (U/L)	33	52	13	12	21	13	15
Total protein (g/dL)	7.0	6.5	7.0	7.3	7.5	7.0	7.4
Albumin (g/dL)	4.2	4.0	4.4	4.5	4.8	4.4	4.4

**Figure 1.**
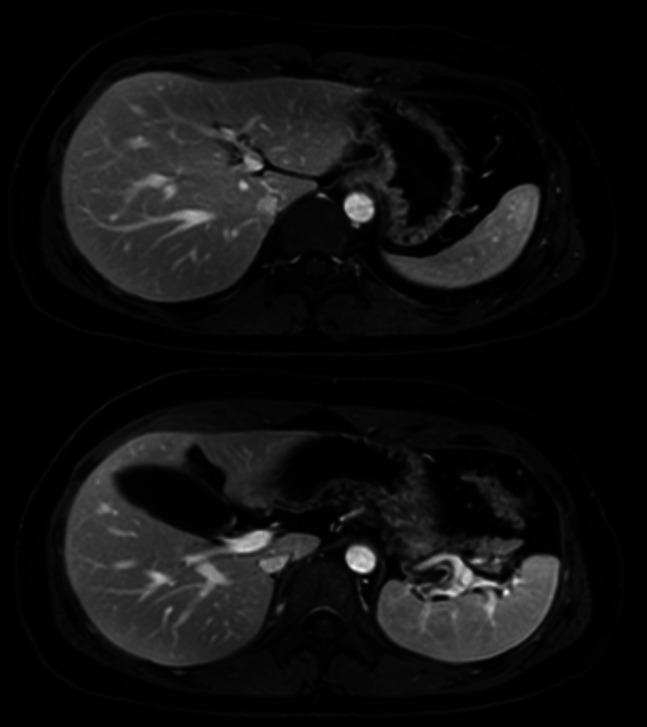
MRI from 2025 demonstrating no signs of cirrhosis, normal sized spleen, and no signs of portal hypertension. Elastography: Calculated mean hepatic stiffness = 1.8 kPa. No findings of steatosis or iron deposition.

In August 2023, liver ultrasound showed geographic steatosis but no fibrosis. He discontinued rifampin, sertraline, and cholestyramine due to side effects and lack of efficacy, continuing only odevixibat, ursodiol, and hydroxyzine. At his last hepatology visit in July 2025, he reported good health and no complaints. Laboratory test results showed TB 0.5 mg/dL, AST 20 U/L, ALT 15 U/L, AP 58 U/L, albumin 4.4 g/dL, MELD-Na 6. Bile acids fractionated and total were normal. MRI elastography showed no fibrosis or portal hypertension (Figure 1).

## DISCUSSION

Since PFIC type 1 has no cure other than liver transplantation, treatment focuses on controlling cholestasis, nutritional deficiencies, and pruritus, similar to other chronic cholestatic conditions.^6^ In this patient, pruritus was the main determinant of poor quality of life. Therefore, a stepwise approach aimed at reducing the bile acid pool was followed.^7^ Initial therapy includes ursodeoxycholic acid,^6,8^ which promotes bile flow through increased bicarbonate secretion by cholangiocytes.^9,10^ If symptoms persist, cholestyramine can help reduce enterohepatic recirculation.^11^

Second line therapy is aimed at increasing the metabolism and excretion of pruritogenic molecules using rifampin.^12^ Additional options include naltrexone,^13^ and sertraline.^14^ Their efficacy has been variable, and the evidence is limited by small samples and heterogeneity.^7,13,15^ Of note, PFIC type 1 is rare and typically described in infants and adolescents, with few cases reported in adults.

Despite maximal medical therapy, he continued to experience debilitating itchiness triggered by exertion. When symptoms remain refractory, bile diversion or liver transplantation may be considered, although these carry notable risks.^16,17^ This patient was not a transplant candidate.

However, he was started on odevixibat, a nonabsorbable ileal bile acid transporter inhibitor along with conventional therapies. Odevixibat selectively blocks the apical sodium dependent bile acid transporter on ileal enterocytes, reducing bile acid resorption.^18^ Although it is approved for patients with PFIC aged ≥3 months (USA), data in adults is limited.^19^ A recent case-series reporting mainly pediatric population reported improved pruritus and sleep after starting odevixibat, with biochemical improvement in most cases. Patients remained on therapy for a median of 11 months. Treatment was well tolerated, with only mild gastrointestinal side effects.^20^

Current guidelines^19^ recommend 40 mcg/kg daily, titrated up to 120 mcg/kg, not exceeding 6 mg/day. Gastrointestinal symptoms are the most frequent side effects,^18^ and this patient only reported self-limited diarrhea.

Odevixibat has been shown to be effective and safe in pediatric randomized clinical trials (RCT).^18^ There are no adult RCT, making this case relevant. Even in the absence of liver dysfunction, pruritus can be profoundly disabling. Odevixibat should be considered in adults with PFIC type 1, and adult RCT are needed to determine long-term outcomes.

## DISCLOSURES

Author contributions: F Gil-Lopez: conceptualization and writing of the original draft, data collection. LA Mercado: IRB application, review of the original draft, data collection. NM Loo: original idea and project conceptualization, project management, manuscript revision from the beginning to the final version. NM Loo is the article guarantor.

Financial disclosures: NM Loo: Ipsen—Advisory Committee/Board Member.

Previous presentation: This case report was presented at the American College of Gastroenterology (ACG) 2025 Annual Scientific Meeting, held October 24–29, 2025, at the Phoenix Convention Center, Phoenix, AZ, USA.

Informed consent was obtained for this case report.

## ABBREVIATIONS:

A1AT: Alpha‑1 Antitrypsink
ALT: Alanine Aminotransferase
AP: Alkaline Phosphatase
AST: Aspartate Aminotransferase
ATPase: Adenosine Triphosphatase
BID: twice daily
MELD: Model for End‑Stage Liver Disease
MELD‑Na: MELD score adjusted for Serum Sodium
MM: normal Alpha‑1 Antitrypsin phenotype
MRI: Magnetic Resonance Imaging
Pa: Kilopascals
PFIC: Progressive Familial Intrahepatic Cholestasis
RCT: Randomized Controlled Trial
